# Lateral Presentation of a Pediatric Thyroglossal Duct Cyst in a Resource-Limited Setting: A Case Report

**DOI:** 10.7759/cureus.111564

**Published:** 2026-06-26

**Authors:** Caleb W Brown, Ana A Moreno-Allen, Ismael Kaitama, Elizabeth M Arze, Andrew C Trecartin

**Affiliations:** 1 Department of Surgery, Quillen College of Medicine, East Tennessee State University, Johnson City, USA; 2 Department of Surgery, Béré Adventist Hospital, Adventist Health International, Béré, TCD; 3 Department of Pathology, Quillen College of Medicine, East Tennessee State University, Johnson City, USA

**Keywords:** global surgery, lateral neck mass, pediatric neck mass, resource-limited setting, sistrunk procedure, thyroglossal duct cyst, ultrasonography

## Abstract

Thyroglossal duct cysts (TGDCs) typically present as midline neck masses in pediatric patients but may rarely present laterally, complicating diagnosis. We report a five-year-old male child with a right-sided lateral neck mass evaluated at a rural district hospital in Chad, where bedside ultrasound demonstrated a complex cystic lesion, and additional imaging was not available. The patient underwent surgical excision with a Sistrunk procedure, and histopathology confirmed an inflamed TGDC. This case highlights the importance of considering TGDC in the differential diagnosis of pediatric lateral neck masses and demonstrates that, in resource-limited settings, clinical assessment and ultrasound may provide sufficient information to support surgical decision-making in select cases.

## Introduction

Thyroglossal duct cysts (TGDCs) represent the most common congenital cervical anomaly and are estimated to affect approximately 7% of the global population, with equal distribution between male and female patients [1]. TGDCs arise from persistence of the thyroglossal duct, an embryologic structure formed during the descent of the developing thyroid gland from the foramen cecum at the base of the tongue to its final position in the anterior neck. This migration occurs between the third and seventh weeks of gestation, during which the thyroglossal duct normally involutes. Failure of this process may result in cyst formation anywhere along the path of thyroid descent [1].

Classically, TGDCs present as painless, mobile midline neck masses near the hyoid bone that characteristically move with swallowing or tongue protrusion. Although often asymptomatic, they may become inflamed or infected, occasionally presenting as abscesses or draining sinuses [1]. Ultrasonography is the preferred initial imaging modality, particularly in pediatric patients, due to its wide availability, low cost, and lack of ionizing radiation or need for sedation. In many cases, ultrasound alone is sufficient to evaluate TGDCs and confirm the presence of normal thyroid tissue. However, computed tomography (CT) and magnetic resonance imaging (MRI) may prove beneficial in select cases in which further diagnostic clarity is needed [1].

While midline presentation is most typical, rare cases of laterally located TGDCs have been reported [2,3]. To our knowledge, only nine cases of TGDCs presenting as lateral neck masses have been reported in the literature across all age groups [2-8]. In such cases, more extensive preoperative imaging may be particularly valuable. We present a rare case of a histopathologically confirmed TGDC presenting as a lateral neck mass in a pediatric patient at a rural district hospital in Chad, highlighting diagnostic and surgical decision-making in a resource-limited setting without access to advanced diagnostic imaging.

## Case presentation

**Figure 1:**
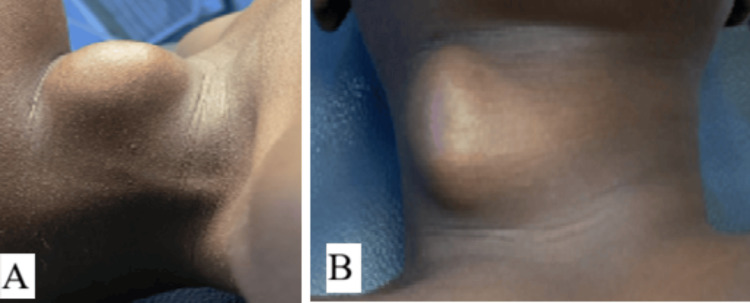
Preoperative clinical photographs demonstrating a right-sided lateral neck mass. (A) Lateral view showing a well-circumscribed, subcutaneous mass located just medial to the right sternocleidomastoid muscle at the level of the thyroid cartilage. (B) Anterior view demonstrating the same lesion, highlighting its lateral position and smooth contour without overlying skin changes

A five-year-old otherwise healthy male child presented to a district hospital with a right-sided neck mass first noticed by his caregivers approximately two months prior. There was no history of preceding infection, trauma, or systemic symptoms. On physical examination, the mass was soft, mobile, and moved with swallowing. It was located just medial to the right sternocleidomastoid muscle at the level of the thyroid cartilage (Figure 1).

**Figure 2:**
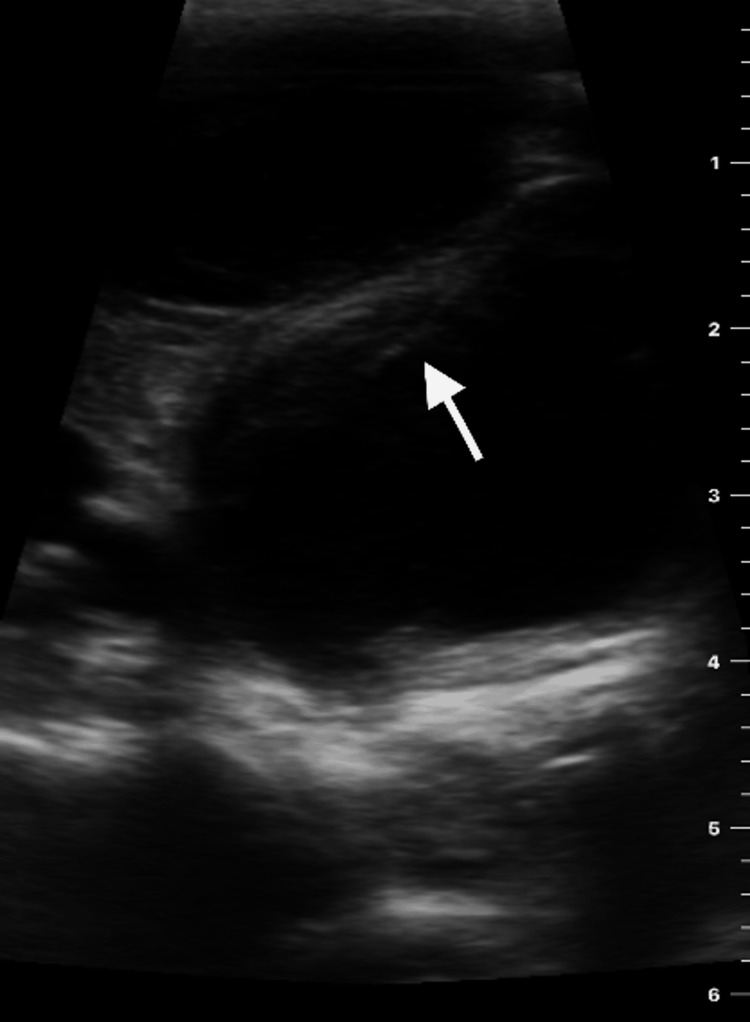
Bedside ultrasonography of the right-sided neck mass demonstrating a superficial, multicystic lesion with internal septations. The arrow indicates a septation between adjacent hypoechoic cystic components, consistent with a complex cystic mass in the right lateral neck

Bedside ultrasound demonstrated a complex, cystic lesion with multiple septations (Figure 2). The thyroid gland appeared normal and was identified in its expected anatomic location. Given the lateral location and cystic nature of the mass, the differential diagnosis included a branchial cleft cyst, lymphatic malformation, or an atypically located TGDC. However, the lesion demonstrated limited compressibility on physical examination, reducing preoperative suspicion for a lymphatic malformation. Additional preoperative cross-sectional imaging was not obtained due to limited availability in this resource-limited setting.

The patient was taken to the operating room for surgical excision. Given the preoperative differential diagnosis, the operation was approached in a manner appropriate for either a branchial cleft cyst or TGDC excision. With the neck in an extended position, a transverse incision was made over the mass. Intraoperatively, the lesion was found to be inflamed and partially fibrotic, making dissection challenging. The mass appeared to have previously ruptured and resealed in areas, with evidence of white granules and inflammatory material extruding into the surrounding soft tissues. It was closely adherent to the right superior pole of the thyroid gland and isthmus.

**Figure 3:**
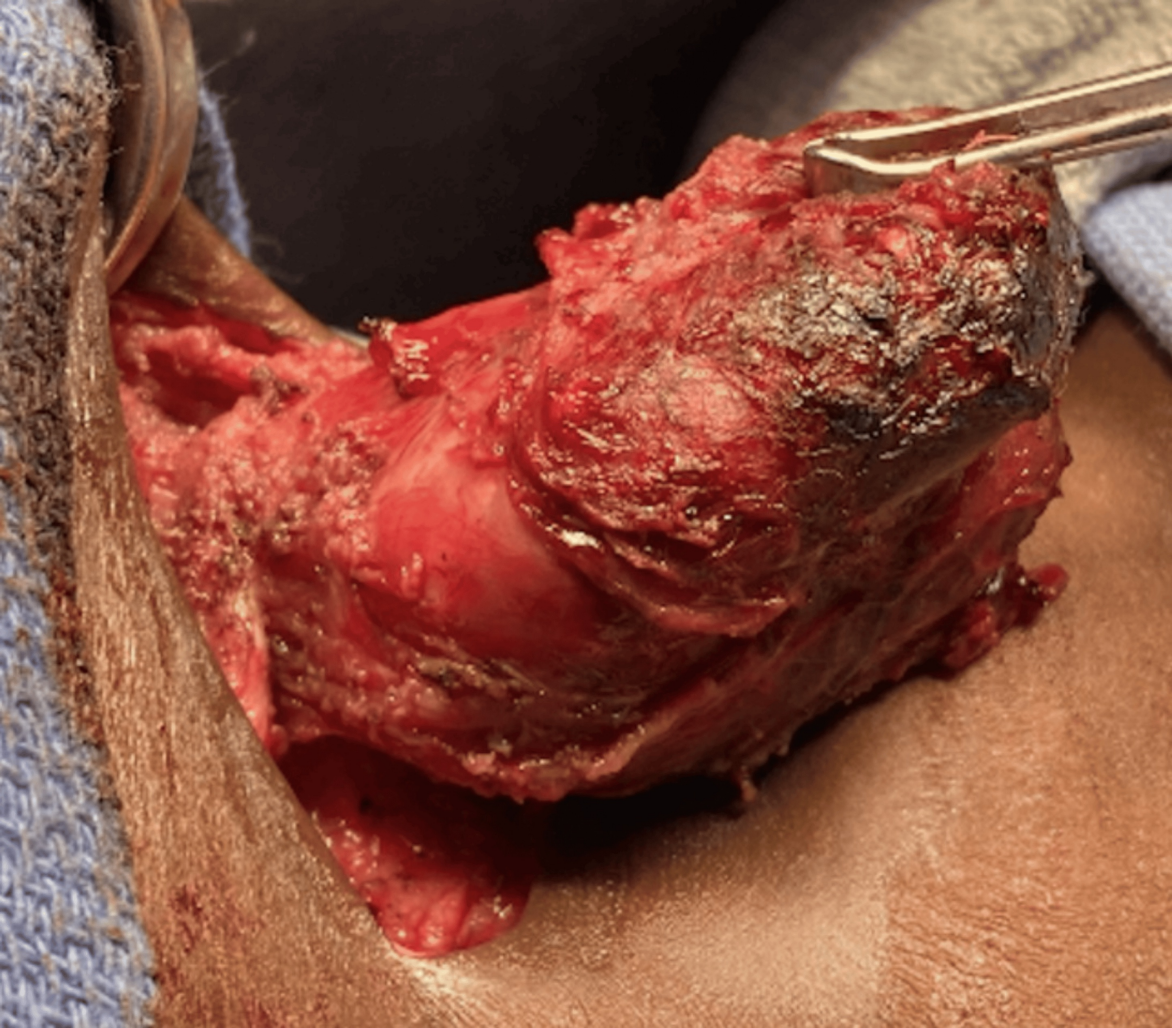
Intraoperative photograph demonstrating a well-encapsulated cystic mass following dissection from the surrounding soft tissues. A retractor positioned beneath the patient's chin (left side of image) provides superior exposure, while an Alice clamp (right side of image) elevates the specimen. The lesion appears inflamed and fibrotic, consistent with the preoperative and intraoperative findings

The mass was carefully separated from the thyroid and surrounding structures using sharp dissection and electrocautery (Figure 3). The presence of normal right and left thyroid lobes was confirmed, and the thyroid gland was preserved. No sinus tract extending into the lateral neck was identified. The lesion was noted to lie lateral to the strap musculature. Dissection continued superiorly to the level of the hyoid bone, where the cyst was found to be connected to the central portion of the hyoid bone. Based on these intraoperative findings, the decision was made to proceed with a formal Sistrunk procedure. En bloc resection of a 2 cm segment of the central hyoid bone, where the mass was firmly adherent. The tract extended less than 1 cm above the hyoid bone, and no sinus tract to the base of the tongue or foramen cecum was identified. The mass was completely excised and sent in formalin for histopathologic analysis. Complete hemostasis was achieved, and the neck incision was closed in layers.

Postoperatively, the patient tolerated oral intake well and reported minimal pain. He was discharged home on postoperative day three. Owing to geographic limitations, postoperative follow-up was conducted regularly via mobile-based communication, which confirmed appropriate healing and favorable clinical outcomes at six months postoperatively.

**Figure 4:**
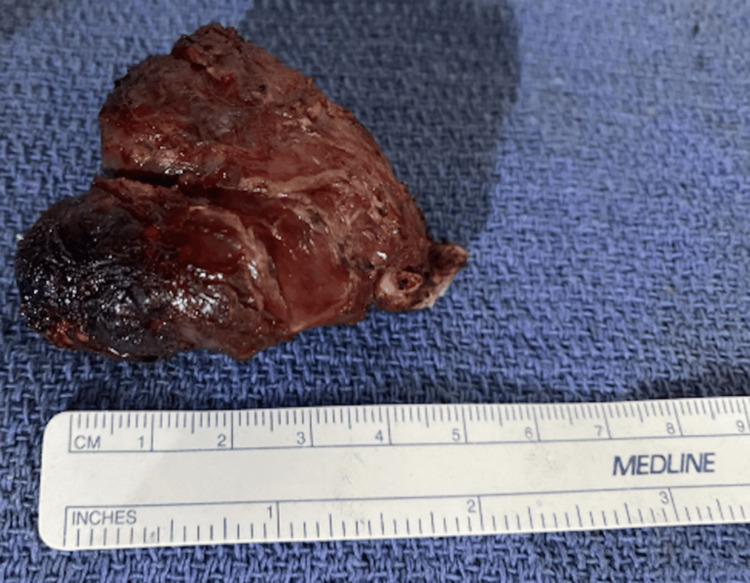
Gross photograph of the excised specimen demonstrating a multilobulated cystic mass measuring approximately 6 cm in greatest dimension. The specimen appears irregular with areas of hemorrhage and fibrosis, consistent with intraoperative findings. A surgical ruler is shown for scale

**Figure 5:**
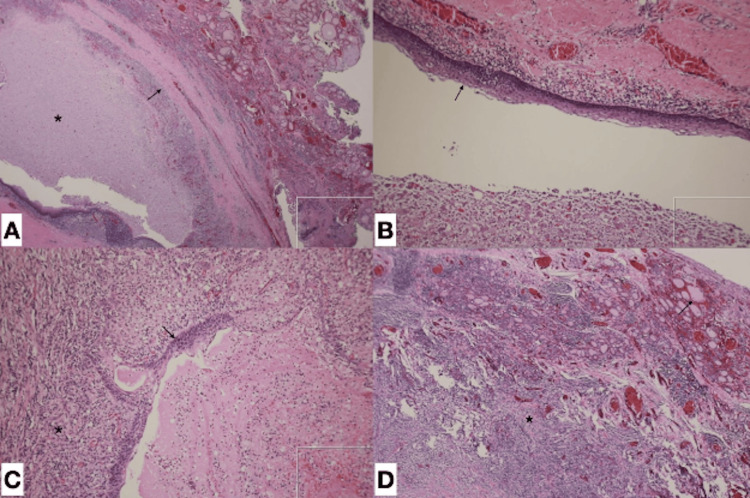
Histopathologic features of a thyroglossal duct cyst (hematoxylin and eosin). (A) Low-power view (2×) demonstrates a cystic structure with a disrupted, inflamed wall (arrow) and luminal eosinophilic debris (asterisk). (B) High-power view (20×) demonstrates an intact benign squamous epithelial lining of the cyst wall (arrow). (C) High-power view (20×) demonstrates a partially denuded squamous epithelial lining (arrow) with adjacent mixed inflammatory infiltrate (asterisk). (D) Low-power view (2×) demonstrates a marked stromal reaction with dense fibrosis and mixed inflammatory infiltrate (asterisk), with adjacent benign thyroid parenchyma showing well-formed follicles containing colloid (arrow)

Gross examination revealed a multilobulated cystic specimen measuring 31 g and 6 × 5 × 3.6 cm, with purple, shaggy soft-tissue fragments (Figure 4). Sectioning demonstrated multiple cavities measuring up to 3.4 cm in greatest dimension, containing tan, homogeneous, gelatinous material. The cyst wall was disrupted in areas, with yellow fibrous tissue and a focal region of calcification. Histopathologic analysis demonstrated a disrupted cyst wall lined by benign squamous epithelium with a surrounding mixed acute and chronic inflammatory infiltrate and marked stromal reaction. Adjacent benign thyroid parenchyma with normal follicular architecture and colloid was identified, consistent with adherent normal thyroid tissue incorporated into the specimen during surgical excision. Overall findings were consistent with an inflamed TGDC (Figure 5).

## Discussion

TGDCs typically present as an anterior midline neck mass near the hyoid bone. Although the most common presentation is around the hyoid bone, they may be found anywhere along the thyroid's embryologic descent [1]. This case is notable for its rare lateral presentation, which complicated the diagnosis, particularly in a resource-limited setting. Only a limited number of laterally presenting TGDCs have been reported in the pediatric population, with most documented lateral cases occurring in adults [4,5].

When evaluating a lateral neck mass in the pediatric population, a TGDC is often low on the differential diagnosis and not initially suspected. Second branchial cleft cysts, the most common branchial cleft anomalies, are classically located along the anterior border of the sternocleidomastoid muscle, making this presentation more suggestive of that diagnosis. Additionally, lymphatic malformations may occur anywhere within the head or neck and can be mistaken for branchial cleft cysts [9]. Cervical lymphadenopathy, including reactive and infectious etiologies, accounts for 38%-45% of pediatric neck masses and may also present in the lateral neck [10]. Given these considerations, a TGDC would not be initially suspected in this case, as the alternative diagnoses are more typically associated with lateral neck presentations.

The workup of a pediatric neck mass typically begins with ultrasound as the first-line imaging modality because it is readily available, cost-effective, and nonionizing [1]. Ultrasound findings can then guide subsequent management, including surgical excision, further imaging with CT or MRI, or observation. Accurate imaging supports preoperative planning and helps tailor the operative approach. For example, while many lateral neck masses are managed with surgical excision, lymphatic malformations are more appropriately treated with sclerotherapy [11]. In cases of TGDCs, surgical management typically includes resection of the central portion of the hyoid bone [1]. When ultrasound does not provide sufficient diagnostic clarity, CT or MRI is recommended to further delineate the lesion and assist with surgical planning in more complex or atypical cases [12]. Based on the history, physical examination, and imaging findings, a presumptive diagnosis can often be made without laboratory studies or preoperative biopsy, as these are unlikely to alter management, and histopathologic evaluation is ultimately obtained from the excised specimen [1].

In this case, a pediatric lateral neck mass was evaluated in a resource-limited setting, adding complexity to the diagnostic process and surgical planning. A bedside ultrasound demonstrated a complex cystic lesion with multiple septations and was interpreted by the operating surgeon due to the absence of radiologists or radiology technologists. Given the previously discussed differential diagnoses and the low TGDC on the differential, additional imaging with CT or MRI could have further characterized this atypical presentation and aided preoperative planning [13]. Specifically, cross-sectional imaging may have improved differentiation between a lymphatic malformation, a branchial cleft cyst, and an atypically located TGDC. Such a distinction could have influenced management decisions, as lymphatic malformations are frequently treated with sclerotherapy rather than surgical excision [11]. However, the absence of advanced imaging did not alter management, as clinical examination and ultrasound findings were sufficient to proceed with surgical excision.

Lateral presentation of a TGDC in the pediatric population is rare, with only a limited number of cases reported in the literature [2-8]. These atypical presentations contribute to diagnostic uncertainty, as such lesions can mimic other lateral neck pathologies, including branchial cleft cysts, lymphatic malformations, and cervical lymphadenopathy. For this reason, additional imaging is often pursued to further characterize the lesion. However, even with advanced imaging, definitive diagnosis is frequently not established until surgical excision and histopathologic evaluation [3,4,7]. In this case, ultrasound, in conjunction with a thorough history and physical examination, enabled the development of a focused differential diagnosis and appropriate surgical planning. As noted, the absence of advanced imaging did not alter the definitive surgical approach.

The Sistrunk procedure remains the standard of care for TGDCs, even in atypical presentations, as it is associated with lower recurrence rates [1,2,4,7]. This case highlights the need to consider a TGDC in the differential diagnosis of lateral neck masses in the pediatric population [2,4,7]. Diagnostic evaluation may be further complicated in resource-limited settings where access to advanced imaging is restricted; however, ultrasound combined with careful clinical assessment can be sufficient to guide appropriate surgical management [13].

## Conclusions

TGDCs most commonly present as midline neck masses but may rarely present in a lateral location, contributing to diagnostic uncertainty. This case highlights the importance of maintaining a broad differential diagnosis when evaluating pediatric lateral neck masses. In resource-limited settings, careful clinical assessment combined with ultrasound may be sufficient to guide appropriate surgical management, even in the absence of advanced imaging. However, this report reflects a single case managed in a specific resource-limited environment, and this approach may not be generalizable to all atypical lateral neck masses. Awareness of atypical presentations can help ensure timely diagnosis and definitive treatment.
